# Characterization and phylogenetic relationships analysis of the complete chloroplast genome of *Capsicum annuum* (Solanaceae)

**DOI:** 10.1080/23802359.2019.1710293

**Published:** 2020-01-14

**Authors:** Mei Bie, Chengdong Han, Xuanyao Wang, Wei Xiao, Kai Song

**Affiliations:** a College of Computer Science and Technology, Jilin University, Changchun, Jilin, P. R. China;; b Institute of Education, Changchun Normal University, Changchun, Jilin, P. R. China;; c School of Life Science, Changchun Normal University, Changchun, Jilin, P. R. China

**Keywords:** *Capsicum annuum*, Solanaceae, chloroplast genome, genome, phylogenetic relationships

## Abstract

*Capsicum annuum* is one of the oldest domesticated crops in the Americas, which is also the most widely grown spice crop in the world. The complete chloroplast genome of *C. annuum* has been assembled and annotated in this paper. Its length was 156,781 bp, containing a large single-copy region of 87,367 bp, a small single-copy region of 17,850 bp, and a pair of IR regions of 25,782 bp in each. The whole chloroplast genome of *C. annuum* contains 135 genes, including 89 protein-coding genes (PCGs), 38 transfer RNA genes (tRNAs), and 8 ribosome RNA genes (rRNAs). The overall nucleotide composition is: A of 30.8%, T of 31.5%, C of 19.1% and G of 18.6%, with a total GC content of the chloroplast genome 37.7% and AT of 62.3%. Phylogenetic relationship analysis was based on 10 plant species using the maximum-likelihood (ML) methods, which showed that the position of *C. annuum* clustered with *C. galapagoense*.

*Capsicum annuum* is an economically important genus of the Solanaceae family (Moscone et al. 2007). It is used are used as vegetable, spice and medicine in the world (Ahn et al. 2016). It has several pharmacological and physiological effects, including analgesic, anti-inflammatory, antioxidant, and anti-obesity properties (Luo et al. 2011). *Capsicum annuum* contains pungent compounds called capsaicinoids. These include capsaicin, dihydrocapsaicin, nordihydrocapsaicin, and trace amounts of other compounds. Capsaicin (trans-8-methyl-N-vanillyl-6-nonenamide) is the major pungent agent, responsible for about 70% of the burn in *C. annuum* (Tomi and Anthony 2019). In this paper, we assembled and annotated the complete chloroplast genome of *C. annuum* and discussed the phylogenetic relationship with other plant species, which provides a valuable resource for genetic resources and gene databases.

The sample of *C. annuum* was collected from the Cucumber-picking garden in Changchun district of Jilin province (Changchun, Jilin, China, 125.41E; 43.92N). The fresh plant tissue and chloroplast (cp) DNA of *C. annuum* were extracted using the modified CTAB method and stored at the Institute of Education, Changchun Normal University (No.IE-CNU-02). The cp DNA was purified and fragmented using the NEB Next Ultra^TM^ II DNA Library Prep Kit (NEB, BJ, and CN) and was sequenced. Quality and adapters control was performed and removed low-quality reads and adapters using the NGS QC Toolkit software (Patel and Jain 2012). The chloroplast genome of *C. annuum* was assembled and annotated using the MitoZ software (Meng et al. 2019). The complete physical map of *C. annuum* chloroplast genome was generated using the OGDRAW version 1.3.1 (Greiner et al. 2019). The annotated complete cp genome of *C. annuum* sequence was deposited in the GenBank with the accession No. NC_018552.1 (Jo et al. 2011).

The complete chloroplast genome of *C. annuum* was a circular in shape with 156,781 bp length, containing a large single-copy region (LSC) of 87,367 bp, a small single-copy region (SSC) of 17,850 bp, and a pair of inverted repeat regions (IRs) of 25,782 bp in each. We annotated and found the cp of *C. sativus* comprised 135 genes, including 89 protein-coding genes (PCGs), 38 transfer RNA genes (tRNAs), and 8 ribosomal RNA genes (rRNAs). The overall nucleotide composition is: 30.8% of A, 31.5% of T, 19.1% of C, and 18.6% of G, with a total G + C content of 37.7% and A + T of 62.3%.

To confirm the phylogenetic position and phylogenetic relationship of *C. annuum* with 10 plant species, the Maximum-Likelihood (ML) method was used. The phylogenetic tree was reconstructed using RaxML version 8.0 (Stamatakis 2014) with the GTR + G + I model. Phylogenetic relationship obtained with the ML approach were identical to those obtained using the Bayesian analysis, which was analyzed by MrBayes version 3.2.5 (Ronquist and Huelsenbeck 2003) and based on the most appropriate model. The phylogenetic tree was performed using MEGA X software (Kumar et al. 2018) by 2000 bootstrap replicates and edited using iTOL version 4.0 (https://itol.embl.de/) (Letunic and Bork 2019). In the phylogenetic tree (Figure 1), the result showed that the chloroplast genome of *C. annuum* is clustered with Solanaceae species of *Capsicum galapagoense* (NC_033524.1) in the phylogenetic relationship.

**Figure 1.**
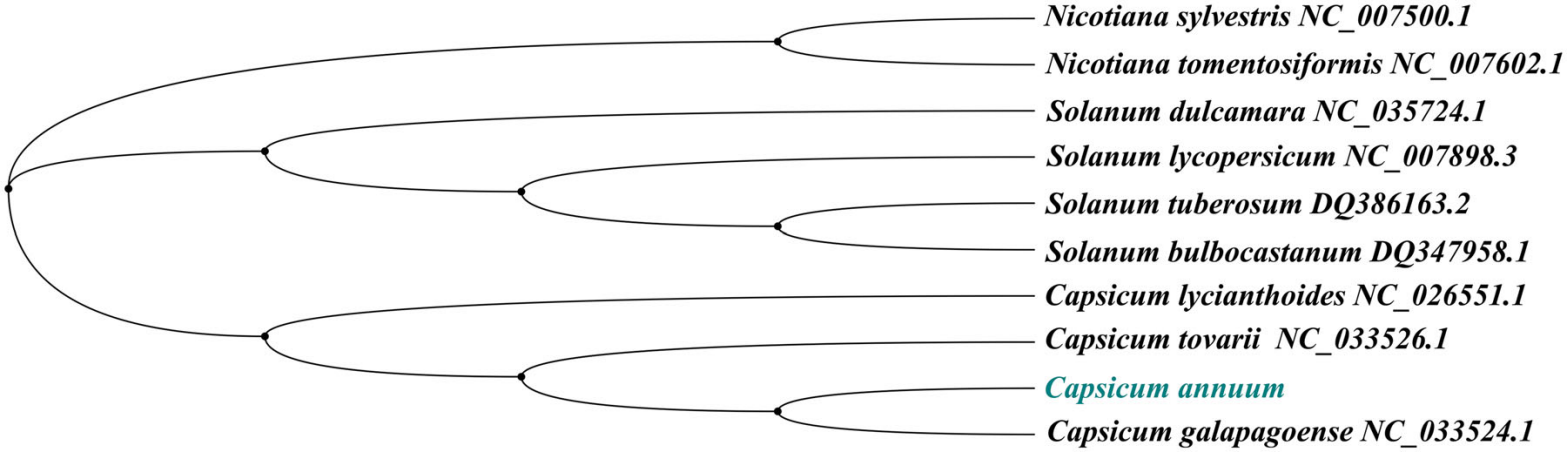
Phylogenetic relationship from 10 species plants chloroplast genome based on the Maximum-Likelihood (ML) analysis of MEGA X. The length of branch represents the divergence distance. All numbers around nodes indicate the bootstrap value from 1000 replicates. All the species chloroplast genomes in this study have been deposited in the GenBank and accession numbers in the figure.
